# Ischemic Strangulation of a Giant Epiploic Appendage through an Omental Defect: a Case Report

**DOI:** 10.5334/jbr-btr.1112

**Published:** 2016-10-24

**Authors:** Tine Hulstaert, An Verena Lerut, Filip Claus, Lieven Van Hoe, Olivier Bladt, Marc Krick

**Affiliations:** 1 OLVZ Aalst, BE

**Keywords:** Abdomen, Epiploic appendagitis, Transomental herniation

## Abstract

We report a rare case of an epiploic appendage twisted through an omental defect, resulting in an epiploic appendagitis at a distance to the colonic wall. The 59-year-old women complained of low abdominal pain and alguria, progressively increasing following a total colonoscopy 4 days earlier.

A 59-year-old women was referred to our emergency department with low abdominal pain and alguria, progressively increasing following a total colonoscopy 4 days earlier. There were no other gastrointestinal complaints or fever. The relevant medical history revealed only an appendectomy. Physical examination showed (rebound) tenderness in the lower abdomen. Laboratory tests revealed a mildly elevated white blood cell count (10330/mL) and elevated C-reactive protein (47 mg/L). Urinary infection was excluded.

Ultrasound showed a large suprapubic mass that was tender when pressure was applied. Contrast-enhanced computed tomography (CT), with peroral and retrograde contrast filling, showed a large fat-containing suprapubic mass of 9.1 × 4 × 5.3 cm (Figures 1A and 1B). The mass seemed to be in contact with the peritoneal cavity through a small hernial defect in the greater omentum, just beneath the transverse colon (Figure 2A and 2B). Inflammation of the surrounding mesenterial fat and thickening of the adjacent peritoneum was present. A small amount of free fluid with a density of 40 HU was seen in the pelvis. Neither pathological bowel dilatation nor wall thickening was present. The diagnosis of a transomental herniation of fatty tissue was presumed.

**Figure 1:**
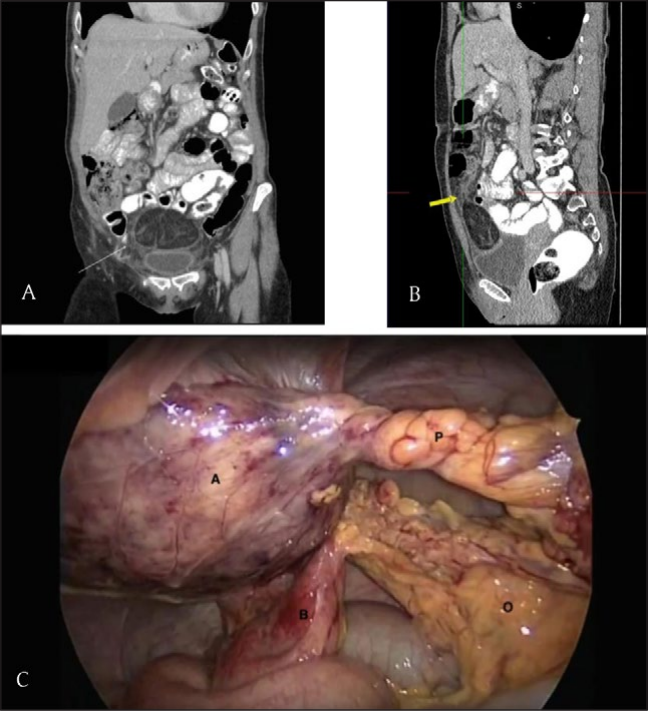
**A** and **B.** Coronal and sagittal CT images of an omental-herniated fatty mass (arrows). **C.** Laparoscopic image of large appendage (A) and its torquated pedicle (P) herniated through the omentum (O). Adhesion of small bowel (B) was present due to inflammation.

**Figure 2:**
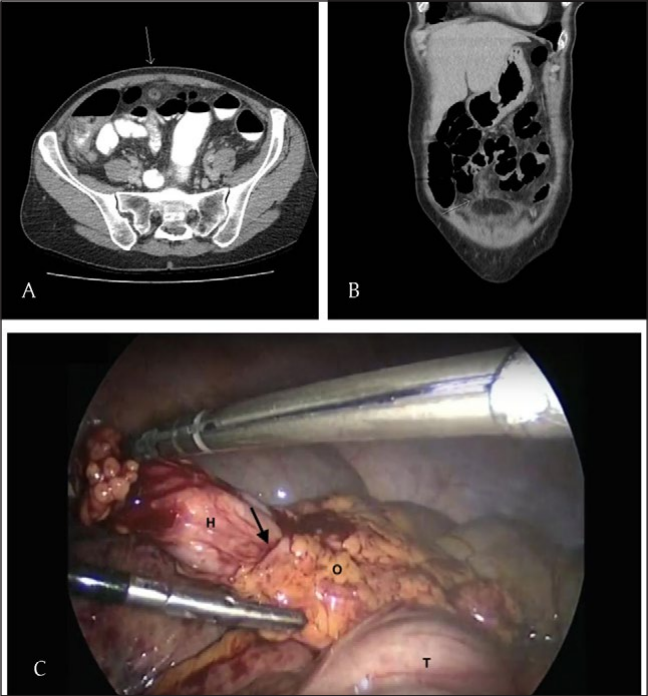
**A** and **B.** Axial and coronal CT images of the orifice in the greater omentum containing the pedicle. **C.** Exploratory laparoscopy shows the herniated pedicle (H) through the defect in the greater omentum (O).

Exploratory laparoscopy revealed a fatty mass, being a giant epiploic appendage of the transverse colon, herniated through a defect in the greater omentum (Figures 1C and 2C). The herniated mass was torquated and strangulated, resulting in an imminent torsion of the transverse colon. The localized inflammatory process had led to adhesion of a small bowel loop (Figure 2C).

Adhesiolysis was performed, the greater omentum was transected and the strangulated fatty mass was resected. Histopathology confirmed a fatty mass of 101 g with fat necrosis surrounded by congestive changes and inflammation, confirming to the diagnosis of a complicated epiploic appendagitis.

## Discussion

Epiploic appendages are pedunculated fatty structures (on average 1–2 cm of thickness and 2–5 cm of length) protruding from the serosal surface of the colon into the peritoneal cavity. Approximately 50–100 appendages are present; most are localized in the caecum and sigmoid along the taenia libera and taenia omentalis. The transverse colon has a smaller number of appendages, as the greater omentum attaches to the taenia omentalis [1,2,8].

A limited blood supply—consisting of a small artery and vein passing through a narrow pedicle—in combination with an excessive mobility makes the appendages prone to torsion, resulting in ischemic or hemorrhagic infarction. Spontaneous thrombosis of the draining vein, resulting in a vascular occlusion, is another cause of primary appendagitis [1,2,3,4,5,8]. Voluminous appendages with a long pedicle in an enlarged peritoneal cavity (e.g., obesity, ascites, recent surgery or delivery) are more prone to twist during heavy exercise or changes in posture [1,4].

Most patients suffering from epiploic appendagitis are between 20 and 60 years of age and complain of pain in the lower abdomen (right or left iliac fossa), as appendages of the caecum and sigmoid are most commonly involved [1,2,8]. There may be some mild fever and leukocytosis, but anorexia, nausea, vomiting, or signs of obstruction are less common [1,2,3]. Physical examination can reveal local tenderness, defense, and rebound. Sometimes a palpable tender mass in the lower abdomen is detected [1]. Clinically, epiploic appendagitis is most often confused with acute diverticulitis and acute appendicitis [7].

Ultrasound typically shows a tender oval-shaped or rounded hyper-echoic mass, surrounded by a hypo-echoic rim and hyper-echoic fat infiltration [2,3,7]. CT findings include a fat-containing lesion near the colonic wall surrounded by an inflammatory rim. A central dot or round or linear hyperdensity, representing a thrombosed vein or hemorrhage, can be seen. Thickening of the parietal peritoneum and compression of adjacent intestinal loops are reported [2,3,5,8]. Differentiating this condition from omental infarction and mesenteric panniculitis may sometimes be difficult [2,3,5].

In most cases, epiploic appendagitis is a self-limiting condition [1,3,4,8]. However, sometimes the inflammation may cause adhesions resulting in obstruction [1]. Epiploic appendagitis presenting within inguinal, femoral, umbilical, or postincisional hernias has been described [1,2,3]. Recently, several cases of a torquated or incarcerated epiploic appendage within a Spigelian hernia have been reported [2,7]. The inflamed epiploic appendages were clearly in direct contact to the bowel wall. This is in contrast to our case, in which the inflammatory process presented at a distance of its attachment to the transverse colon because of a transomental herniation. This type of herniation is very rare [6]. The increased distance of the inflamed mass to the colon combined with the herniation made the preoperative diagnosis less obvious. Given the signs of peritonitis and the presumed diagnosis of transomental herniation, an explorative laparoscopy was justified.

## Conclusion

Epiploic appendagitis should always be included in the differential diagnosis of a patient presenting with acute abdominal pain. In this case report, we described a rare presentation of torquated epiploic appendagitis at a distance to the colonic wall, secondary to a transomental herniation.

## References

[1] Ghahremani GG, White EM, Hoff FL (1992). Appendices epiploicae of the colon: Radiologic and pathologic features. RadioGraphics.

[2] Capaccio E, Di Vito L, Derchi LE (2011). Epiploic appendage torse within a Spigelian hernia: US and CT findings. Journal of Clinical Ultrasound.

[3] Özkurt H, Kratag O, Karaarslan E (2007). Clinical and CT findings of epiploic appendagitis within an inguinal hernia. Diagnostic and Interventional Radiology.

[4] Jeanmonod P, Sperling J, Seidel R (2011). Torquated giant appendix epiploica mimicking intraperioneal liposarcoma: Report of a case. Int Surg.

[5] Pereira JM, Sirlin CB, Pinto PS (2005). CT an MR imaging of extrahepatic fatty masses of the abdomen and pelvis: Techniques, diagnosis, differential diagnosis, and pitfalls. RadioGraphics.

[6] Camera L, De Gennaro A, Longobardi M (2014). A spontaneous strangulated transomental hernia: Prospective and retrospective multi-detector computed tomography findings. World J Radiol.

[7] Coulier B, Broze B (2010). Epiploic appendagitis within a Spigelian hernia. Journal of the Belgian Society of Radiology.

[8] Coulier B (2010). Contribution of US and CT for diagnosis of intraperitoneal focal fat infarction (IFFI): A pictorial review. Journal of the Belgian Society of Radiology.

